# Characterization of the *Ghd8* Flowering Time Gene in a Mini-Core Collection of *Miscanthus sinensis*

**DOI:** 10.3390/genes12020288

**Published:** 2021-02-19

**Authors:** Zhihui Guo, Meilan Xu, Hironori Nagano, Lindsay V. Clark, Erik J. Sacks, Toshihiko Yamada

**Affiliations:** 1 Graduate School of Environmental Science, Hokkaido University, Sapporo, Hokkaido 060-0810, Japan; guozhihui-2008@hotmail.com; 2 Field Science Center for Northern Biosphere, Hokkaido University, Sapporo, Hokkaido 060-0811, Japan; xumeilan0506@gmail.com (M.X.); nagano2@fsc.hokudai.ac.jp (H.N.); esacks@illinois.edu (E.J.S.); 3 Department of Crop Sciences, University of Illinois, Urbana-Champaign, Urbana, IL 61801, USA; lvclark@illinois.edu; 4 Global Center for the Research of Food, Land and Water Resources, Hokkaido University, Sapporo, Hokkaido 060-8589, Japan

**Keywords:** *Miscanthus sinensis*, *Ghd8*, gene expression, flowering time, sequence diversity, geographic distribution

## Abstract

The optimal flowering time for bioenergy crop *Miscanthus* is essential for environmental adaptability and biomass accumulation. However, little is known about how genes controlling flowering in other grasses contribute to flowering regulation in *Miscanthus*. Here, we report on the sequence characterization and gene expression of *Miscanthus sinensis* *Ghd8*, a transcription factor encoding a HAP3/NF-YB DNA-binding domain, which has been identified as a major quantitative trait locus in rice, with pleiotropic effects on grain yield, heading date and plant height. In *M. sinensis*, we identified two homoeologous loci, *MsiGhd8A* located on chromosome 13 and *MsiGhd8B* on chromosome 7, with one on each of this paleo-allotetraploid species’ subgenomes. A total of 46 alleles and 28 predicted protein sequence types were identified in 12 wild-collected accessions. Several variants of *MsiGhd8* showed a geographic and latitudinal distribution. Quantitative real-time PCR revealed that *MsiGhd8* expressed under both long days and short days, and *MsiGhd8B* showed a significantly higher expression than *MsiGhd8A*. The comparison between flowering time and gene expression indicated that *MsiGhd8B* affected flowering time in response to day length for some accessions. This study provides insight into the conserved function of *Ghd8* in the Poaceae, and is an important initial step in elucidating the flowering regulatory network of *Miscanthus*.

## 1. Introduction

The genus *Miscanthus* is a rhizomatous, self-incompatible, C4 perennial grass that has a natural distribution from the tropics to ~50° N in East Asia and Oceania [1], including *Miscanthus sinensis*, *Miscanthus floridulus* and *Miscanthus sacchariflorus*, and is closely related to sugarcane (*Saccharum officinarum*) and sorghum (*Sorghum bicolor*). Owing to its environmental adaptability, *Miscanthus* is used as forage for livestock feed, as an ornamental for landscapes, and as a bioenergy crop that provides high yields with low nutrient requirements [2,3]. For *Miscanthus* production, optimization of flowering time is essential to obtain high biomass yield under different environments [4,5], and may also impact biomass quality [6]. Controlling flowering also assists intra- and interspecific hybridizations between *Miscanthus* and *Saccharum* to facilitate the introgression of genes for disease resistance and abiotic stress tolerance from *Miscanthus* to sugarcane [7]. Additionally, to develop seed-based hybrid cultivars of *Miscanthus*, uniform flowering of the parental genotypes will be needed, and this has the potential to reduce the cost of establishment and accelerate domestication relative to the current standard approach of vegetatively propagating rhizomes of *Miscanthus× giganteus*, which is a hybrid between *M*. *sacchariflorus* and *M. sinensis*, and has recently attracted considerable attention as a feedstock crop for bioenergy [8,9].

*Miscanthus* has a long life span, exceeding 15 years, and it typically flowers annually [10], indicating that it has a complex mechanism for renewed vegetative growth after flower initiation. Initially, *M. sinensis* was described as a day neutral plant by Deuter [11], whereas Jensen et al. [8] demonstrated that flowering regulation in *M. sinensis* was complex, operated by thermal time/degree days but also a photoperiod sensitivity mechanism. In their latest study, Dong et al. [7] reported that *M. sinensis* was a facultative short-day (SD) plant, and photoperiod strongly affected *Miscanthus* flowering. Thus, it would be desirable to study the photoperiod regulation of flowering time in *M. sinensis*. The major regulatory genes for photoperiod control of flowering have been evolutionarily conserved in flowering plants but their specific effects can vary greatly among genera and species [12]. To date, the photoperiod regulation of flowering has been extensively investigated in the SD plant rice (*Oryza sativa*), and two independent genetic pathways have been identified [12]. One is the rice *OsGI-Hd1-Hd3a* pathway, which has been conserved in the SD plant sorghum [13], and is orthologous with the *GI*-*CO*-*FT* pathway in the long-day (LD) plant *Arabidopsis* [12]. In rice, *GIGANTEA (GI)* upregulates *HEADING DATE 1 (Hd1),* the ortholog of *CONSTANS (CO),* which regulates the expression of *HEADING DATE 3a* (*Hd3a*) to promote flowering in SD and delay flowering in LD [14,15,16]. Another flowering time pathway is *Ghd7*-*Ehd1*-*Hd3a,* which has been found in rice, sorghum and maize (*Zea mays*) but is absent from *Arabidopsis thaliana* [12,17,18,19,20]. *GRAIN NUMBER, PLANT HEIGHT AND HEADING DATE 7* (*Ghd7*) is a grass-specific regulator of flowering and related traits. In rice, *Ghd7* represses flowering under LD by down-regulating *EARLY HEADING DATE 1* (*Ehd1*) and *Hd3a* [19]. In SD, *Ehd1* activates *Hd3a* expression and induces floral transition [17,21]. *Ehd1* is regulated by many genes, including *Hd1*, *GI*, *Ghd7*, *PSEUDORESPONSE REGULATOR PROTEIN 37 (PRR37*), and *GRAIN NUMBER, PLANT HEIGHT AND HEADING DATE 8 (Ghd8)* [19,22,23,24,25]. In sorghum, there is a similar but not identical flowering time pathway. Sorghum *CENTRORADIALIS 15 (SbCN15)*, the sorghum ortholog of rice *Hd3a* (*FLOWERING LOCUS T, FT*), may modify flowering time in a photoperiod-insensitive manner [18,26,27]. *SbCO* acts as an activator of flowering in SD by inducing the expression of *SbEhd1*, *SbCN8* and *SbCN12* (*FT*-like genes), whereas in LD, *SbCO* activity is inhibited by *SbPRR37* [13]. *SbPRR37 [Maturity1(Ma1)]* and *SbGhd7 (Ma6)*, which are promoted by sorghum *PHYTOCHROME B (SbPhyB),* inhibit flowering by decreasing the expression of *SbEhd1, SbCN8* and *SbCN12* under LD [18,26,27]. *Ma2* delayed flowering in LD by selectively enhancing the expression of *SbPRR37* and *SbCO* [28].

To date, information on the genetics of flowering regulation in *Miscanthus* is in its infancy [4,7,8]. Genetic linkage maps revealed fourteen flowering time quantitative trait loci (QTLs) in *Miscanthus* [29,30,31]. Dong et al. [29] found one *Miscanthus* flowering QTL on LG02 that corresponded to sorghum maturity gene *Ma3* (*PhyB*) [26] and another located on LG01 that corresponded to the *ASYMMETRIC LEAVES-like1* gene, which controls proximal–distal patterning in *Arabidopsis* petals [32]. Gifford et al. [30] found a *Miscanthus* QTL that corresponded to sorghum maturity gene *Ma5* (*PHYTOCHROME C, PhyC)*. Jensen et al. [31] reported eleven flowering QTLs on LG04 in *M. sinensis,* three of which were robust QTLs related to the age-dependent flowering pathway (*SQUAMOSA PROMOTER BINDING PROTEIN-LIKE* and MADS-box *SEPELLATA2*) and the gibberellin pathway (gibberellin-responsive bHLH137). However, the functions of these candidate flowering time genes in the *Miscanthus* QTLs have yet to be verified, and allelic sequence variation for these genes has yet to be described. At present, *Hd1*/*CO* is the only candidate flowering time gene that has been screened in *Miscanthus* for sequence diversity and its geographic distribution, with large differences found among accessions from the Asian mainland relative to those from the Japanese archipelago [33].

Recently, *Ghd8* (*DTH8/LHD1/Hd5/LH8*) has been found to be a key regulator of the *Ghd7-Ehd1-Hd3a* pathway in rice [34]. *Ghd8* was initially identified as *HAP3b* in *Arabidopsis*, which can promote flowering in *Arabidopsis* by enhancing the expression of key flowering time genes, such as *FT* and *SUPPRESSOR OF OVEREXPRESSION OF CONSTANS1 (SOC1)*, under LD [35]. In rice, *Ghd8* has a dual function to inhibit flowering under LD and promote flowering under SD by regulating *Ghd7*, *Ehd1*, *RICE FLOWERING LOCUS T 1 (RFT1)* and *Hd3a* [22,23]. In particular, *Ghd8* encodes a protein transcription factor, heme activator protein 3 (HAP3)/ nuclear factor-YB (NF-YB), that in rice binds to CCAAT motif in the promoter region of *Ghd7*, as part of a complex with HD1 and OsHAP5b [34]. In rice, a 19 bp deletion in *Ghd8* causes a loss-of-function that confers early flowering and thus adaptation to high latitudes; this allele is widely distributed among cultivars from Northern China and Japan [36,37], and has been selected and used widely for breeding early heading varieties in Hokkaido [37]. Therefore, *Ghd8* plays a key role in the domestication and adaptation of rice in Hokkaido. It is worthwhile to investigate if a similar process occurred in *Miscanthus* during its migration northward after the last glacial maximum. *Ehd1* in rice is induced by blue light in the morning, and *Ghd7* suppression of *Ehd1* is induced by red light in the morning under LD, thereby suppressing flowering, whereas under SD, the peak of *Ghd7* expression shifts to night, and this misaligned timing allows *Ehd1* to induce *Hd3a* and promote flowering [12]. Genomic synteny and collinearity are common features in the Poaceae [38,39], and have also been confirmed among rice, sorghum, switchgrass and *M. sinensis* genomes [29,31,40,41,42,43]. Previous studies have identified genes/QTLs under parallel evolution across grass species [31,33,44,45,46,47,48]. To date, there have been no reports of *Ghd8* in C4 bioenergy crops such as sorghum, switchgrass and *Miscanthus*. Thus, a key question this study seeks to answer is the following: does *M. sinensis* have a functional *Ghd8* that contributes to the regulation of flowering time? Moreover, we expect that if *Ghd8* regulates flowering in *M. sinensis*, the gene’s expression in the day will follow a pattern of differential flowering times under LD relative to SD. In this study, we cloned the ortholog of *OsGhd8* in a mini-core collection of *M. sinensis* with the aim to 1) characterize allelic and deduced amino acid sequence diversity and geographic distribution, and 2) determine expression patterns in response to photoperiod and relate these to previously obtained data on days to first flower under LD and SD. 

## 2. Materials and Methods

### 2.1. Plant Materials and Growth Conditions

Twelve Miscanthus accessions (clones maintained by vegetative propagation) were studied for gene sequence variation and expression over time in response to two photoperiod treatments (15 h, LD; 12.5 h, SD) (Table 1). The twelve accessions included eleven *M. sinensis* from known locations in China and Japan, representing latitudes ranging from 18° N to 45° N, and one M. floridulus from 20.9° S in New Caledonia (we consider *M. floridulus* conspecific with *M. sinensis* [49,50] and hereafter refer to the entire panel as *M. sinensis*). The M. sinensis accessions represent six genetic groups that were previously identified by Clark et al. [49,50]. Dong et al. [7] previously evaluated the same twelve accessions for days to first flowering under day lengths of 15, 12.5 and 10 h in controlled environment chambers, and observed strong flowering time responses that varied by latitude of origin. In the current study, six pots of each accession were established by planting rhizomes in 2 L plastic pots containing soilless medium consisting of compost, vermiculite, calcined clay and peat moss (Forex Mori Sangyo Co., Ltd., Hokkaido, Japan) and growing these in a greenhouse at Hokkaido University in Sapporo, Japan (43.1° N, 141.3° E), with natural photoperiod. 

After 40 d of establishment in the greenhouse, the *Miscanthus* plants were cut to 5 cm above the soil surface and moved into growth chambers (BioTRON LH-350S, NK Systems, Nippon Medical & Chemical Instruments Co., Ltd., Osaka, Japan) under constant long days (15 h). Pots were rotated randomly inside and between the chambers on a daily basis to minimize between-chamber and within-chamber environmental effects. The growth chambers provided 400 ± 50 μmol m^−2^ s^−1^ of photosynthetically active radiation with fluorescent lamps (Hitachi FLR40S-EX-N/M/36-A, Hitachi, Ltd., Tokyo, Japan), as measured with a quantum sensor (MIJ-14PARII, Environmental Measurement, Fukuoka, Japan). After 30 d of establishment in the chambers, the plants were subjected to one of two day-length treatments: LD (15 h light/9 h dark) and SD (12.5 h light/11.5 h dark), with three pots per accession given LD and three given SD. The temperature in the chambers was a constant 23 °C for the duration of the experiment. At planting and again at the start of each experiment, 15 g of 12-9-12 compound fertilizer (Kumiai Grassland No. 8; Hokkaido Fertilizer Co., Ltd., Japan) was added to each pot. Irrigation was provided to each pot each day. At day 38, one week after commencement of the LD or SD treatment, the three topmost leaves from each of the three pots per accession within each treatment were harvested and pooled at Zeitgeber times (ZT) of 3, 9, 15 and 21 h for one 24 hour light–dark cycle. 

### 2.2. Genomic DNA Extraction and Isolation of Ghd8 in Miscanthus

Genomic DNA was isolated from young, healthy leaves by the modified cetyltrimethylammonium bromide (CTAB) [51] protocol using the DNeasy Plant Mini Kit (Qiagen, Tokyo, Japan) according to the manufacturer’s instructions. Gene-specific primers (Forward primer 1: 5′-GAAAGGCGATTAAGAGGAGAAT-3′; Forward primer 2: 5′-CACCATAAGCTAGCTGACTAGCT-3′; Reverse primer 1: 5′-GCAAGTATCGTTTGTCGTCGTCTT-3′) for *Ghd8* were designed by aligning multiple sequences retrieved from the *Miscanthus sinensis* v7.1 genome [41] and its close relative sorghum using the *Sorghum bicolor* v3.1 genome from Phytozome v.13 (https://phytozome-next.jgi.doe.gov (accessed on 15 September 2019)). Amplification of *Ghd8* was accomplished by polymerase chain reactions (PCRs) containing 30 ng of total genomic DNA as a template and LA Taq polymerase (TaKaRa Bio, Shiga, Japan). Amplification conditions were 1 min at 95 °C, followed by 30 cycles of 30 s at 95 °C, 30 s at suitable primer temperature and 1 m 30 s at 72 °C. PCR products were separated on 0.8% agarose gels by electrophoresis. Amplified bands of desired molecular weight were eluted from the agarose gel with the NucleoSpin^®^ Gel and PCR Clean-up kit (Macherey-Nager GmbH & Co. KG, Düren, Germany) and cloned into a pGEM-T Easy vector (Promega, Madison, WI, USA) using the TA-Blunt Ligation Kit (Nippon Gene Co., Ltd., Toyama, Japan) following the manufacturer’s instructions. Positively transformed colonies were selected on blue-white selection on ampicillin/IPTG/X-Gal LB plates, and plasmids were purified using a High Pure Plasmid Isolation Kit (Roche, Sigma-Aldrich, Tokyo, Japan). About 20 plasmid clones of each genotype were sequenced in both directions with a BigDye Terminator v3.1 Cycle Sequencing Kit (Applied Biosystems, Foster City, CA, USA) via an ABI PRISM 3130 Genetic Analyzer (Life Technologies, Carlsbad, CA, USA) according to the manufacturer’s instructions. To identify true alleles and to limit the potential for misidentifying point mutations and indels resulting from PCR and sequencing errors as true alleles, we set a quality-control threshold of at least three colonies with the identical sequence for inclusion in further analysis and reporting.

### 2.3. RNA Isolation and Quantitative Reverse Transcription-PCR Analysis

Leaves were sampled from fully expanded healthy leaves at ZT 3, 9, 15 and 21 h in the growth chamber. All samples were immediately frozen in liquid nitrogen and stored at −80 °C until analysis. Total RNA was isolated from frozen leaves with a Favorgen^®^ Plant Total RNA Extraction Mini Kit (Favorgen Biotech Corp., Taiwan) and treated with DNase I (TaKaRa Bio, Shiga, Japan) to remove contaminating genomic DNA. cDNA was synthesized from purified RNA using an oligo (dT) 20 primer and random hexamer primers with Invitrogen™ M-MLV Reverse Transcriptase (Invitrogen, Carlsbad, CA, USA) according to Dwiyanti et al. [52]. The transcript levels for candidate genes were determined by quantitative real-time PCR (qRT-PCR). The PCR reactions (20 μL) contained 4.6 μL of the cDNA synthesis reaction mixture diluted to 1/15th of its original volume, 5 μL of 1.2 μM primer premix, 0.4 μL ROX Reference Dye (50×) and 10 μL of TB Green^®^ Premix Ex Taq™ II (Tli RNaseH Plus) (TaKaRa Bio, Shiga, Japan). Expression levels were determined on a StepOnePlus™ Real-Time PCR System (Applied Biosystems, Foster City, CA, USA) with cycling conditions of 95 °C for 5 min, followed by 40 cycles of 95 °C for 10 s, 60 °C for 20 s and 72 °C for 30 s. Values were normalized to *ACTIN* (Misin17G008500) as an internal control. A reaction mixture without reverse transcriptase was also used as a control to confirm the absence of genomic DNA contamination. Amplification of a single DNA fragment was confirmed by melting-curve analysis of quantitative PCR and gel electrophoresis of the PCR products. Relative changes in gene expression were estimated following the 2^−ΔΔCt^ method [53]. Averages and standard errors of relative expression levels were calculated for three independently synthesized cDNAs. The forward primer used for *ACTIN* (Misin17G008500) gene expression was 5′-AGGGCTGTTTTCCCTAGCATCGT-3′, and the reverse primer was 5′-GGGTACTTGAGCGTGAGAATACCTC-3′. Primers were designed for *MsiGhd8* based on the putative functional alleles. The forward primer used for *MsiGhd8A* (Misin13G040800) gene expression was 5′-CTCAACCGCTACCGCGAGGTC-3′, and the reverse primer was 5′- TCATCCGCCGCGCCATCT-3′. The forward primer used for *MsiGhd8B* (Misin07G127500) gene expression was 5′-ACGTCGGGCTCATGATGGGAGCA-3′, and the reverse primer was 5′-ATACGACTTCCGTGCTGCCGT-3′.

### 2.4. Data Analysis

The nucleotide sequences were assembled with ATGC v.6 software (GENETYX Co., Tokyo, Japan). *O. sativa*, *S. bioclor*, *M. sinenesis* genome sequences (Phytozome v.13, 100 kb) spanning *Ghd8* gene were used for microsynteny /collinearity analysis, which was determined and visualized by Genome Evolution Analysis (GEvo) (http://genomevolution.org/CoGe/GEvo.pl (accessed on 2 January 2021)) and the high-resolution sequence analysis tool from the Accelerating Comparative Genomics (CoGe) toolkit (http://genomevolution.org/CoGe/ (accessed on 2 January 2021)). Multiple alignments of nucleotide and amino acid sequences were implemented in MEGA X [54,55,56], using ClustalW [57] with default settings. Phylogenetic trees were generated in MEGA X [54,55] using the Neighbor-Joining (NJ) method [58] with the substitutional model of Kimura 2-parameter [59]. The corresponding sequences of rice and sorghum were used as an out-group. Support for each node was tested with 1000 bootstrap repetitions [60]. The trees were edited and visualized in FigTree ver.1.4.4 (http://tree.bio.ed.ac.uk/software/figtree/ (accessed on 5 September 2020)). Relative changes in mean ± standard error of the mean (SE) gene expression were analyzed in Microsoft Excel (Microsoft Office 2016, Microsoft Inc., Seattle, WA, USA) and then exported to GraphPad Prism 9 software (GraphPad Software, San Diego, CA, USA) for visualization. Statistical tests for differences among means were conducted by a Student’s *t*-test or analyses of variances (ANOVAs) using GraphPad Prism 9 software (GraphPad Software, San Diego, CA, USA). The DNA sequences obtained are available from DDBJ (http://www.ddbj.nig.ac.jp/index-e.html (accessed on 21 December 2020)) with the accession numbers LC598392 to LC598437.

## 3. Results

### 3.1. Characterization of M. sinensis Ghd8

**Figure 1:**
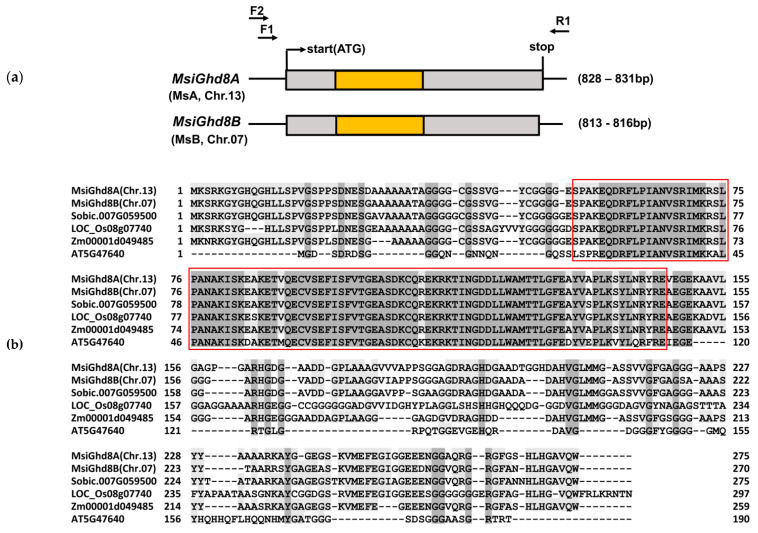
Gene structure and multiple alignment analysis of *Miscanthus sinensis Ghd8* homoeologs and their comparison with orthologs from four other plant species. (**a**) Gene structure of *MsiGhd8A* and *MsiGhd8B.* F, forward primer; R, reverse primer; the primer pairs F1/R1 and/or F2/R1 were used to detect open reading frames (ORFs) for *Ghd8*. The start codon (ATG) and stop codon (TGA) are highlighted in black. The yellow box represents the HAP3/NF-YB domain. (**b**) Multiple amino acid sequence alignments for Ghd8 from *M. sinensis* (this study), *Sorghum bicolor* (Sobic.007g059500), *Oryza sativa* (LOC_Os08g07740), *Zea mays* (Zm0001d049485) and *Arabidopsis thaliana* (AT5G47640). The HAP3/NF-YB domain is boxed in red. The *M. sinensis* sequence used for alignment was from accession PMS-382.

**Figure 2:**
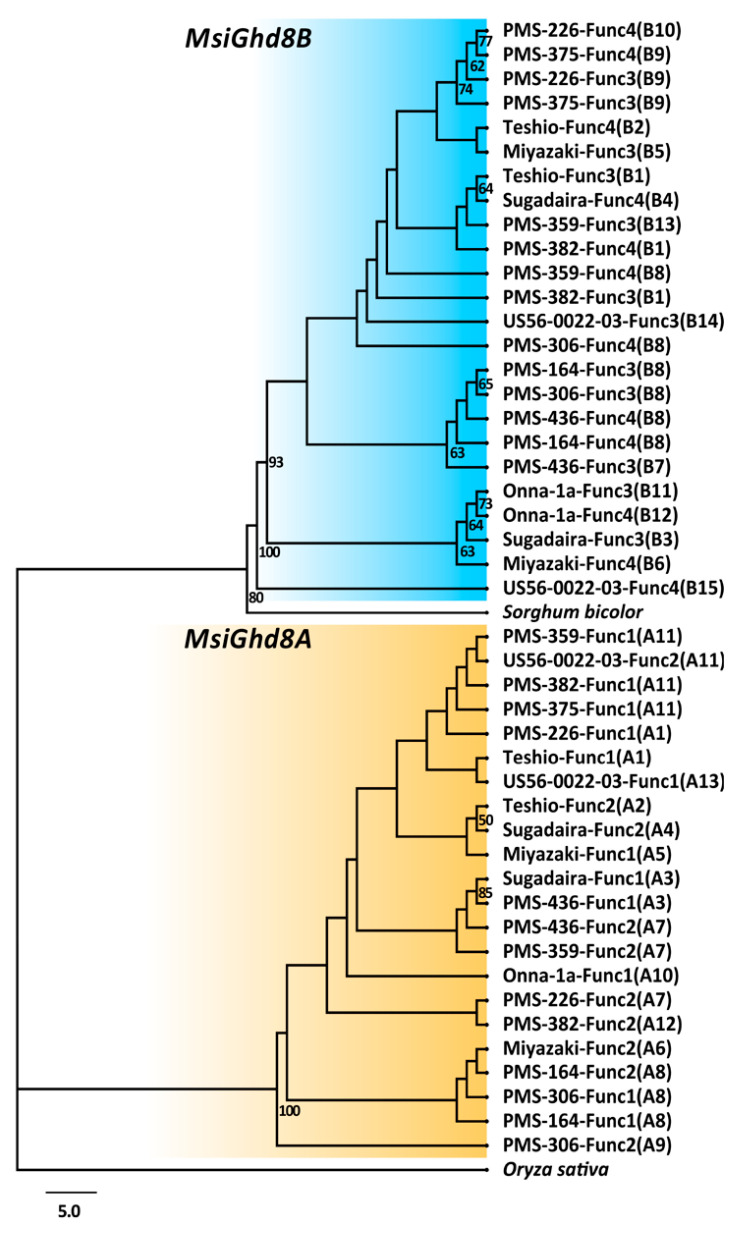
Phylogenetic tree inferred by neighbor-joining method for nucleotide sequences of 42 Ghd8 alleles from 11 accessions of *Miscanthus sinensis* and four alleles from one *Miscanthus floridulus* accession. *Sorghum bicolor* (Sobic.007g059500) and *Oryza sativa* (LOC_Os08g07740) were used as an out-group. The phylogenetic tree was divided into two clusters, which were classified as *MsiGhd8A* and *MsiGhd8B*, one for each of the two subgenomes. Bootstrap values for nodes supported in >50% of 1000 bootstrap replicates are shown. Allele names with A or B prefix indicate putatively functional alleles types based on predicted amino acid sequence variants, which are named in parentheses and correspond to the names in Figure 3 and Figure S2, Table 1 and Table S1.

In *M. sinensis*, two homoeologous *Ghd8* loci, *MsiGhd8A* located on chromosome 13 (Chr.13) and *MsiGhd8B* on chromosome 7 (Chr.07), were identified, with one on each of this paleo-allotetraploid species’ subgenomes (Figure 1). A total of 46 *MsiGhd8* alleles were identified from the 12 wild-collected *M. sinensis* accessions (Figure 2 and Table S1). Sequence alignment indicated that the ORF lengths of *M. sinensis Ghd8* ranged from 813 to 831 nucleotides, and contained one exon that coded for 270 to 276 amino acid residues (Figure 1). Multiple sequence blasting in Phytozome v13 (https://phytozome-next.jgi.doe.gov (accessed on 20 March 2020)) revealed that the nucleotide sequences of *M. sinensis Ghd8* were highly similar to those in other plant species, such as *S. bicolor* (Sobic.007G059500, 88.6–92.3%), *O. sativa* (LOC_Os08g07740, 72.2–73.3%), *Z. mays* (Zm0001d049485, 82.7–86.3%) and *A. thaliana* (AT5G47640, 32.0–32.9%). A microsynteny assessment of genomic regions adjacent to *Ghd8* in rice, sorghum and *M. sinensis* identified four colinear genes, including *Ghd8*, aligned with the same relative genomic order in a 100 kbp region, which was consistent with the identification of LOC_Os08g07740 as an ortholog of rice *Ghd8* [22] (Figure S1 and Table S2). Therefore, based on sequence similarity and gene collinearity, two homoeologous *Ghd8* loci in *M. sinensis* were designated as orthologs of *Ghd8* in rice and sorghum, and probable orthologs of *HAP3b* in *A. thaliana*. Neighbor-Joining (NJ) phylogenetic trees revealed a robust separation of clades representing *MsiGhd8A* (22 alleles) and *MsiGhd8B* (24 alleles) (Figure 2). The phylogenetic trees indicated that the sorghum *Ghd8* was more similar to *MsiGhd8B* than *MsiGhd8A*. Two accessions (Onna-1a and PMS-375, 16.7%) were homozygous at the *MsiGhd8A* locus, and all accessions were heterozygous at the *MsiGhd8B* locus (Table 1). Pairwise DNA sequence comparisons showed that the similarity of *MsiGhd8A* open reading frame (ORFs) ranged from 98.7% (Teshio -Func2 vs. PMS-226 -Func2, Teshio -Func2 vs. PMS-382 -Func2, Teshio -Func2 vs. US56-0022-03 -Func2) to 100% (Sugadaira -Func1 vs. PMS-436 -Func1, PMS-164 -Func1 vs. PMS-306 -Func1, PMS-375 -Func1 vs. PMS-382 -Func1) (Table S1). Similarly, the nucleotide sequence similarity of *MsiGhd8B* ORFs varied from 97.9% (Sugadaira -Func4 vs. US56-0022-03 -Func4) to 100% (PMS-164 -Func3 vs. PMS-306 -Func3, PMS-164 -Func4 vs. PMS-436 -Func4, PMS-226 -Func3 vs. PMS-375 -Func3) (Table S1). 

**Figure 3:**
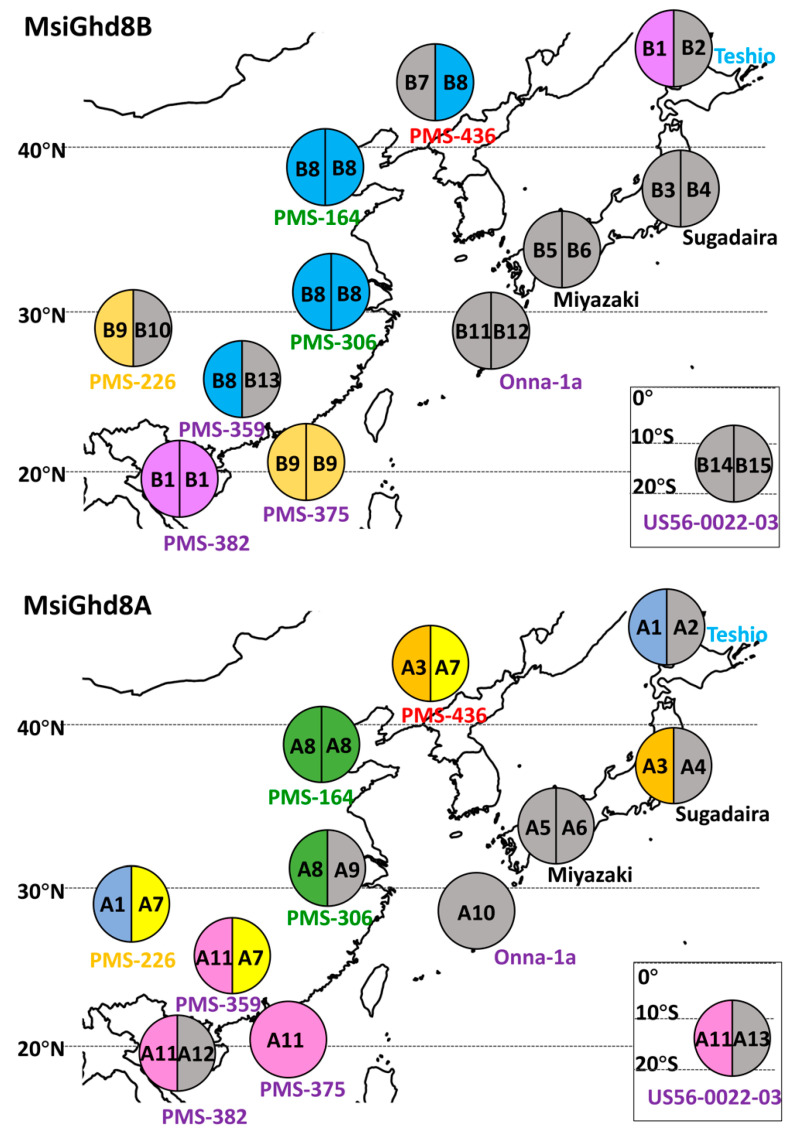
Geographical distribution of MsiGhd8A and MsiGhd8B predicted amino acid sequence variant types in *Miscanthus sinensis*. Pie charts with one to two sections represent the number of detected alleles. A or B prefix indicates putatively functional alleles types based on predicted amino acid sequence variants, corresponding to the names in Figure 2, Table 1 and Table S1. Different colors in pie charts represent different variant types that occurred in more than one accession; variant types that were observed only once have a gray background, corresponding to Table S1. Accessions’ names were colored to represent *M. sinensis* genetic groups previously described by Clark et al. [49,50]; Sugadaira and Miyazaki were changed from yellow to black for making the map clear.

Comparison of the 46 *MsiGhd8* alleles derived from the 12 wild-collected *M. sinensis* accessions in this study with the alleles in the *Miscanthus sinensis* v7.1 genome [41] revealed 35 non-synonymous single nucleotide variants (nsSNVs), 36 synonymous single nucleotide variants (sSNVs) and two 3-bp insertions in ORFs, with some accessions having more than one SNV per allele (Table S1). Considering the fact that the nucleotide diversity cannot exactly represent the protein diversity owing to synonymous SNVs in ORFs, Ghd8 protein variant types were analyzed in the present study (Table 1 and Table S1, Figure 2 and Figure 3). Accounting for nsSNVs, 13 predicted amino acid sequence types of MsiGhd8A and 15 of MsiGhd8B (28 total) were identified from the 12 *M. sinensis* accessions (Table 1 and Table S1, Figure 2 and Figure 3). The amino acid sequence similarity of putatively functional MsiGhd8A and MsiGhd8B variants ranged from 92.1% to 94.2%. Notably, the deduced amino acid sequence of Ghd8 in *M. sinensis* indicated that the gene products contain a HAP3/NF-YB DNA-binding domain located from position 53 to 146 (Figure 1b), which is critical for the transcription factor function of *Ghd8* gene products. Though no putatively non-functional alleles were detected, four nsSNVs in the HAP3/NF-YB DNA-binding domain of MsiGhd8 (two in *MsiGhd8A* and two in *MsiGhd8B*) were observed in five accessions, with one nsSNV of *MsiGhd8A* found in each of two accessions (Sugadaira and PMS-436), one nsSNV of *MsiGhd8A* found in Teshio and one nsSNV of *MsiGhd8B* in PMS-226 and another nsSNV of *MsiGhd8B* found in US56-0022-03 (Table S1).

### 3.2. Geographical Distribution of Naturally Occurring MsiGhd8 Protein Variants

Some of the MsiGhd8 protein variants were found over a broad geographic range, whereas others had restricted patterns of occurrence (Table 1, Figure 2, Figure 3 and Figure S1). In the A subgenome, variant A1 was the most broadly distributed, with occurrence in accessions that originated from the mid and highest latitudes in this study (PMS-226 from Sichuan basin and Teshio from northern Hokkaido Japan), but it was infrequently observed (16.7% of accessions). In contrast, A7 was distributed widely and the second-most frequently observed variant (25% of accessions). A3 was limited to two accessions, one in Northern China and one in Central Japan; however, DNA sequence analysis indicated that A3 and A7 are closely related (Table S1) and thus represent a broadly distributed group in mainland Asia and Japan. A11 had a restricted distribution from New Caledonia to Guangdong China with a latitude ranging from 20.9° S to 22.9° N and was the most frequent variant (33.3% of accessions) but was absent from mid and high latitudes in mainland Asia and Japan. However, phylogenetic analysis of the DNA sequence revealed that A11 and A1 protein variants were closely related and thus represented a widely distributed group from east to west and from north to south. A8 was limited to mid latitudes in mainland Asia. The other eight variants were each observed in only one accession. A2 and A3, which encode one additional amino acid resulting from the same 3-bp insertion in the nucleotide sequence, were limited to Northern Japan and China. 

In the B subgenome, variant B1 was observed from Hainan to Hokkaido but infrequently (16.7% of accessions). In mainland Asia, B8 was also broadly distributed from low to high latitude and frequent (25% of accessions). B9 was observed in two accessions, one in Sichuan Basin and one in Southern China. The other twelve variants were each observed in only one accession. Phylogenetic analyses of DNA sequence indicated the following closely related protein variant groups: B7 and B8; B9 and B10; B1, B4 and B13; B3, B6, B11 and B12 (Figure 2 and Figure 3).

### 3.3. Expressions Patterns of M. sinensis Ghd8

**Figure 4:**
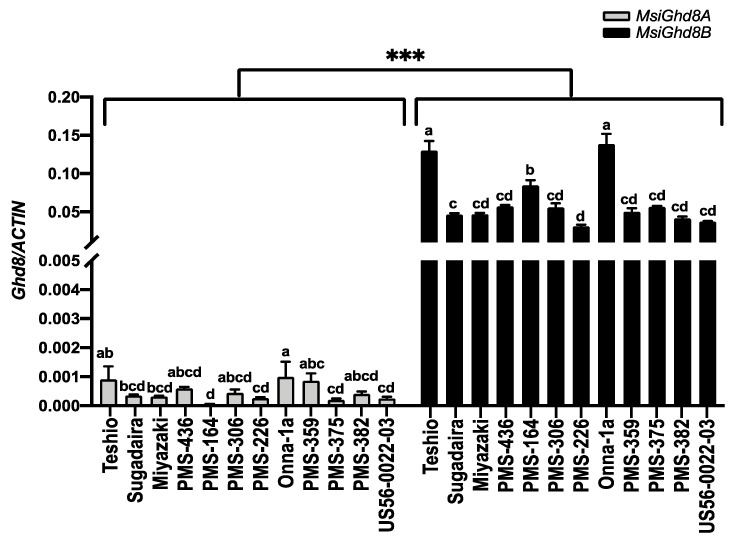
Expression of *MsiGhd8* at Zeitgeber time 9 for 12 *Miscanthus sinensis* accessions under long days (15 h). Grey and black represent *MsiGhd8A* and *MsiGhd8B*, respectively. Relative mRNA levels are expressed as the ratios to *ACTIN* transcript levels. Mean ± 1SE for three replications are given for each data point. A different letter on top of a bar indicates significant difference between accessions within each subgenome according to the Tukey HSD (95% family-wise confidence level) multiple comparison tests. *** shown between the two subgenomes indicates a significant difference at *p* < 0.001 according to the Student’s *t*-test.

**Figure 5:**
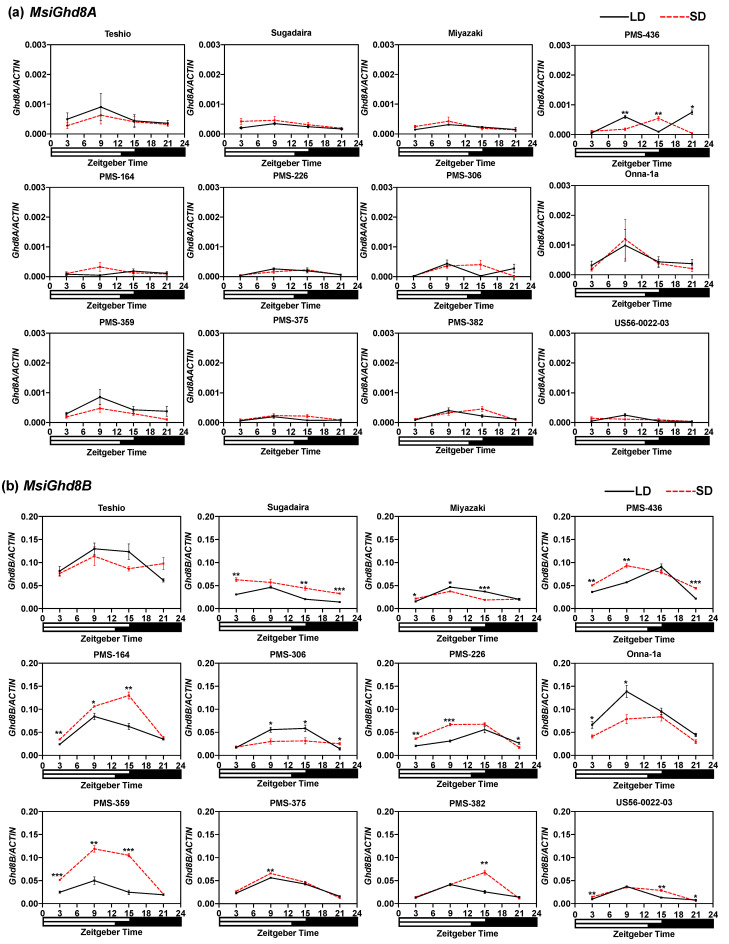
Diurnal expression of *MsiGhd8* in 12 *Miscanthus sinensis* genotypes under long days (15 h, long-day (LD); solid black lines) and short days (12.5 h, short-day (SD); red dashed line). (**a**) *MsiGhd8A* and (**b**) *MsiGhd8B*. Relative mRNA levels are expressed as the ratios to *ACTIN* transcript levels. The numbers below the x-axis indicate Zeitgeber times (ZT) of the day. The white bar at the bottom of each graph indicates the light period and the black bar indicates the dark period. Mean ± 1SE for three replications are given for each data point. Asterisks indicate significant difference between the two means under LD and SD at the same ZT of the day (Student’s *t*-test, * *p* < 0.05, ** *p* < 0.01, *** *p* < 0.001). No asterisk indicates the difference between the two means is not statistically significant (*p* < 0.05).

For each of the *M. sinensis* accessions, expression of *Ghd8* (assessed as the ratio of *Ghd8*/*ACTIN* mRNA transcript abundance) from the B subgenome was one to two orders of magnitude greater than for the A subgenome (Figure 4 and Figure 5). Within each subgenome, large differences among the accessions for *Ghd8* expression were observed (Figure 4 and Figure 5). The two accessions with the highest morning-expression of *MsiGhd8B* under LD (Teshio and Onna-1a) also had the highest expression of *MsiGhd8A* (Figure 5). Interestingly, under LD, Onna-1a was the latest flowering of the accessions, but Teshio was the earliest flowering, and neither flowered under SD. Three patterns of diurnal *MsiGhd8* expression were observed: day peak, night peak and no clear peak (Figure 5). The most common diurnal *MsiGhd8* expression pattern observed was a day peak at ZT9 or ZT15 (Figure 5), which is later than the dawn peak that has been reported for rice, suggesting that optimal timing may differ between *M. sinensis* and rice. For the B subgenome, the LD/SD ratio of *Ghd8* expression at ZT9, was >1 for three accessions, <1 for five accessions and ~1 for four accessions (Figure 5). Notably, two of the accessions with *MsiGhd8B* LD/SD ratios ~1 also had a relatively low expression, were from the tropics (PMS-382 and US56-0022-03) and were among the only three accessions in the panel that did not flower under LD (Figure 5, Table 1); the third accession (PMS-375) was similar, with a small but significantly lower expression under LD than SD at ZT9. In contrast to the B subgenome, the A subgenome LD/SD ratio of *Ghd8* expression at ZT9 was >1 for only one accession (PMS-436) and ~1 for eleven accessions.

## 4. Discussion

The results of the current study demonstrate that *Ghd8* is present in *M. sinensis*, and likely contributes to a regulatory function for flowering time in this species in a manner that is similar to that in rice. Firstly, collinearity analysis revealed that two homoeologous *Ghd8* loci (Misin13G040800 and Misin07G127500), one each in the two *M. sinensis* subgenomes (MsA and MsB), corresponded to the same genomic region on rice Chr.08 (LOC_Os08g07740) and sorghum Chr.07 (Sobic.007g059500) (Figure S1 and Table S2), which was consistent with the known paleo-duplications (rice Chr.08- sorghum Chr.07, sorghum Chr.07- *Miscanthus* Chr.13 and Chr.07) from the ancestral grass chromosomal groups [29,40,41]. Additionally, at each of the two homoeologous *Ghd8* loci in *M. sinensis*, each accession in this study had at least one putatively functional full-length allelic copy containing a highly conserved HAP3/NF-YB DNA-binding domain that is required for the transcription factor function of *Ghd8* in rice [22] and *A. thaliana* [61]. Moreover, the two homoeologous *Ghd8* loci in *M. sinensis* expressed and may have a conserved function to regulate flowering time. If the *M. sinensis Ghd8* genes were non-functional, we would expect a high frequency of accessions to have no functional alleles due to a lack of selection pressure, but this was not observed. Moreover, the *M. sinensis Ghd8* genes were highly expressed (especially from the B subgenome), which is a necessary requirement for function. 

Perhaps the strongest evidence for *Ghd8* having a role in regulating the photoperiod-sensitive induction of flowering in *M. sinensis* comes from the observed differences in the gene’s expression under LD relative to SD during the day and its relationship to observed days to first flower among the accessions. If the critical time for *Ghd8* to suppress *Ehd1* via *Ghd7* is in the morning, as was reported for rice [34], or at ZT9 for *M. sinensis*, as evidenced by a frequent peak at that time, then an LD/SD *Ghd8* expression ratio >1 would be expected to delay or prevent flowering under LD and hasten flowering under SD, whereas a LD/SD ratio <1 would be expected to do the opposite (i.e., hasten flowering under LD and delay or prevent flowering under SD). A value of one for the day LD/SD *Ghd8* expression ratio would indicate that *Ghd8* did not regulate flowering time in that accession, and that other genes conferred any observed differences in flowering time associated with day length. For the *M. sinensis* B subgenome, four of the eight accessions with ZT9 LD/SD *Ghd8* expression ratios differing from ~1 had values that were consistent with their observed flowering times (Table 1, Figure 5). Two of these four accessions (Miyazaki and PMS-306) had ZT9 LD/SD *MsiGhd8B* expression ratios >1 and flowered substantially earlier under 12.5 than 15 h day length, similar to the short-day (SD) response reported for rice [22,23]. The other two accessions (PMS-436 and PMS-164) had ZT9 LD/SD *MsiGhd8B* expression ratios <1 and flowered early under 15 h but failed to flower under 12.5 h day lengths; at ZT 3, both accessions also had LD/SD *MsiGhd8B* expression ratios <1 and a third accession, Sugadaira, performed similarly with an LD/SD ratio <1 at ZT3 but not at ZT9. Notably, the five accessions with day LD/SD *MsiGhd8B* expression ratios that were consistent with their flowering times were among the six most northerly accessions (≥29.9° N) in the panel (only Teshio was not included), suggesting that *MsiGhd8B* regulation of flowering time may predominate in *M. sinensis* from high latitudes. The three tropical accessions with *Ghd8* expression ratios ~1 uniquely did not flower under 15 h but did flower under 12.5 h day length, suggesting that this adaptation was conferred not by *Ghd8* but some other, yet to be determined gene(s). Given that grasses have multiple pathways to regulate flowering time, including two known major pathways for photoperiod regulation of flowering time that each has multiple modifiers, we would not expect every accession in the panel to have its flowering time predominantly conferred by any one gene, including *Ghd8*. Nevertheless, we identified a signal of *Ghd8* regulation of flowering time from nearly half of the *M. sinensis* accessions in the panel.

In contrast to the B genome, two lines of evidence suggest that the *M. sinensis* A genome homoeolog of *Ghd8* does not substantially contribute to the photoperiod regulation of flowering time. First, eleven of the twelve accessions in the panel had ZT9 LD/SD *MsiGhd8A* expression ratios ~1, yet all the accessions studied had different flowering time responses to LD or SD. Second, the expression of *MsiGhd8A* was substantially lower than the expression of *MsiGhd8B* for each accession. The lower expression observed for *MsiGhd8A* than *MsiGhd8B* was consistent with a previously observed *M. sinensis* genome-wide expression bias in favor of the B subgenome, with ~10% more pairs of genes having higher expression in the B subgenome [41]. Thus, *MsiGhd8A* may be a case of reduced or neo-functionalization, which is common in organisms with duplicated genomes [62].

The four nsSNVs identified in the HAP3/NF-YB domain of MsiGhd8 from five accessions (Table S1) could have an important effect on protein stability and function. Though the sample size is limited, it is worthwhile to consider what role these variants might have in regulating the flowering time of *M. sinensis*. If the *M. sinensis Ghd8* functions similarly to the rice *Ghd8*, by regulating *Ghd7* as part of a complex with HD1 and HAP5b [34], then there is the potential for reduced stability of the complex to affect the phenotype. Complex formation, such as Ghd8-OsHAP5b-Hd1, and DNA-binding are stochastic processes that can be affected by the concentration of the molecules involved. For example, if expressed copies of *Ghd8* that have a non-functional or reduced-functioning DNA binding site produce protein molecules that remain able to form a complex with the products of HD1 and HAP5b, then they may compete with copies of *Ghd8* that have fully functional DNA binding sites, thereby reducing the quantity of functional complex and consequently reducing the transcription of *Ghd7* and promoting flowering. Similarly, copies of *Ghd8* that have a conserved DNA binding site, but which can form a complex that has an unstable conformation, may not be able to promote *Ghd7* transcription, yet may compete for *Ghd7* binding sites with molecules of the complex that can act as a functional transcription factor. In domesticated rice, non-functional alleles of *Ghd7*, *Ghd8* and *Hd1* enabled early flowering and thus the expansion of cultivation to high latitudes for food production [63], whereas for undomesticated *M. sinensis*, natural selection appears to have resulted in functional alleles *Ghd8* and *Hd1* [33], conferring adaptation to high latitudes. For *M. sinensis Hd1*, a high frequency of non-functional alleles differentiated accessions from the Japanese archipelago and with those from mainland Asia [33], which is different from what we observed for *Ghd8* in the current study. 

Further research is needed to quantify the effects of individual putative functional *MsiGhd8* alleles with nsSNVs and/or sSNVs on flowering time in response to day length. These studies can evaluate segregating populations derived from controlled biparental crosses, or be achieved by gene editing. The current study provides information on which alleles are present in different accessions that can be used to conduct genetics studies of segregating biparental populations. Additionally, the sequence data obtained by the current study for many different natural *MsiGhd8* alleles can be used to plan gene-editing studies in *Miscanthus*, rice or other species to dissect function while controlling for genetic background.

Dong et al. [7] observed that short days (<12.5 h) were also a signal for *M. sinensis* from high latitude plants to induce a short-internode dormancy response, which is an adaptation to protect apical meristems from damaging low temperatures during winter in high latitudes, and this dormancy response was epistatic to flowering. Similar dormancy responses to short days have been found in several quantitative short-day, perennial, C4 grasses, including *M. sacchariflorus* [4], switchgrass (*Panicum virgatum*) [64] and big bluestem (*Andropogon gerardii)* [65]. Additional research is needed to determine whether *MsiGhd8* also mediates dormancy directly or indirectly.

In *Arabidopsis* and rice, extensive studies have revealed the underlying genetic mechanisms for regulating heading date. Using yeast and animal systems, it has been demonstrated that HAPs, a CCAAT-box-binding transcription factor, form a heterotetramer or heterotrimer for transcription activation. In *A. thaliana*, *HAP3b* subunits can directly interact with *Hd1*/*CO* through its CCT-domain, forming CCAAT-binding CBF complexes that bind to *FT* promoters and activate transcription to promote flowering under LD [66,67]. In rice, the grass-specific gene *Ghd7* is upregulated by a Ghd8-OsHAP5b-Hd1 complex under LD, enabling *Ghd7* to suppress *Ehd1* and delay flowering [36,63,68,69,70,71,72]. However, *Hd1*/*CO* also competes with the complexes to promote *Hd3a*/*RFT1* expression, creating a tradeoff relationship for photoperiod sensitive flowering under SD conditions. Thus, the regulatory network controlling flowering time is complex and quantitative, which likely accounts for the great plasticity of this trait in diverse populations. Whether MsiGhd8 protein can bind these flowering-related gene products (*Hd1*/*CO* and *Ghd7*) forming NF-Y complexes as described in rice remains to be confirmed in future studies, but the results of the current study suggest it is likely. 

In addition to flowering time, *Ghd8* has been found to regulate multiple developmental and physiological processes in rice. In previous studies, *OsGhd8* has been associated with stress tolerance and regulation of photosynthesis [23,73,74]. *OsGhd8* up-regulated *MONOCULM 1* (*MOC1*), a key gene controlling tillering and branching; this increased the number of tillers and primary and secondary branches [23]. Wang et al. [73] found a cis-regulatory variation in the *Ghd8* promoter, associated with cold tolerance, thus contributing significantly to the ecological adaptation of rice varieties to high latitudes. Adachi et al. [74] indicated that *CARBON ASSIMILATION RATE 8* (*CAR8*), identical to *DTH8*/*Ghd8*/*LHD1*, affected multiple physiological aspects relating to photosynthesis in rice, such as CO_2_ assimilation rate and hydraulic conductivity. Given the great allelic diversity observed for *M. sinenesis Ghd8* in the current study, it would be desirable to determine if this gene also regulates a range of important physiological and developmental traits of *Miscanthus*.

In summary, this study identified two homoeologous loci of *MsiGhd8* among a mini-core collection of *M. sinensis*, with one on each of this paleo-allotetraploid species’ subgenomes. Several alleles and predicted amino acid sequence variants of *MsiGhd8* showed a geographic and latitudinal distribution. The gene expression of *MsiGhd8* correlated with the flowering date for some accessions in response to the photoperiod. The diverse *MsiGhd8* expression patterns illustrated the complicated flowering regulatory network in *Miscanthus*. Further studies will be necessary to clarify the molecular mechanism of regulatory networks of flowering-related genes in *Miscanthus*, and to potentially improve biomass yield and quality by the regulation of the reproductive phase. 

## Figures and Tables

**Table 1:** Provenance, flowering time under short or long days and amino acid sequence diversity for two homoeologous *Ghd8* loci in a mini-core panel of 11 *Miscanthus sinensis* and one *Miscanthus floridulus* accessions.

Genotypes	Ploidy	Lat	Long	Genetic Group †	Genetic Group Color Code †	Days to First Flowering ‡		Variant Types Classified by Predicted Protein in *Ghd8* Homoeologs			
						12.5 h	15 h	MsiGhd8A		MsiGhd8B	
*M. sinensis* “Teshio”	2x	44.9	141.9	Northern Japan	Blue		66	A1	A2	B1	B2
*M. sinensis* “Sugadaira”	2x	36.0	138.1	Southern Japan	Yellow		96	A3	A4	B3	B4
*M. sinensis* “Miyazaki”	2x	31.8	131.4	Southern Japan	Yellow	61	167	A5	A6	B5	B6
*M. sinensis* “PMS-436”	2x	41.3	123.7	Korea/North China	Red		115	A3	A7	B7	B8
*M. sinensis* “PMS-164”	2x	37.3	114.3	Yangtze-Qinling	Green		130	A8	A8	B8	B8
*M. sinensis* “PMS-306”	2x	29.9	118.8	Yangtze-Qinling	Green	84	173	A8	A9	B8	B8
*M. sinensis* “PMS-226”	2x	26.6	106.8	Sichuan Basin	Orange	76	189	A1	A7	B9	B10
*M. sinensis* “Onna-1a”	2x	26.5	126.8	SE China plus tropical	Purple		274	A10		B11	B12
*M. sinensis* “PMS-359”	2x	22.9	112.3	SE China plus tropical	Purple	81	179	A11	A7	B8	B13
*M. sinensis* “PMS-375”	2x	19.6	110.3	SE China plus tropical	Purple	142		A11		B9	B9
*M. sinensis* “PMS-382”	2x	18.9	109.5	SE China plus tropical	Purple	184		A11	A12	B1	B1
*M. floridulus* “US56-0022-03”	2x	−20.9	165.3	SE China plus tropical	Purple	114		A11	A13	B14	B15

† *M. sinensis* genetic groups determined by Clark et al. [49,50]. ‡ Days to first flowering under short days (12.5 h) or long days (15 h) by Dong et al. [7]; empty cells indicate flowering did not occur. A or B prefix indicates putatively functional alleles types based on predicted amino acid sequence variants in the A and B subgenomes, respectively, corresponding to Figures 2 and 3, Table S1. Empty cells of MsiGhd8A indicated that only one allele type was detected in Onna-1a and PMS-375, and therefore, these two accessions were homozygous at *MsiGhd8A*.

## Data Availability

All data necessary for confirming the conclusions of the article are present within the article, Figures and Tables, and within Supplementary Tables and Figures.

## Supplementary Materials

The following are available online at https://www.mdpi.com/2073-4425/12/2/288/s1. Figure S1: Chromosome organization of *Ghd8* gene orthologous regions (100 kbp) from *Oryza sativa*, *Sorghum bicolor* and *Miscanthus sinensis*. Figure S2: Geographical distribution of *MsiGhd8A* and *MsiGhd8B* alleles in *Miscanthus sinensis*. Table S1: A summary of polymorphic sites in *MsiGhd8* open reading frames (ORFs) from 12 *Miscanthus sinensis* accessions. Table S2: Colinear genes near orthologs of *Ghd8* in *Miscanthus sinensis*, *Sorghum bicolor*, and *Oryza sativa*.
