# Supplementary material for: Characterization of the Ghd8 Flowering Time Gene in a Mini-Core Collection of Miscanthus sinensis
Source: Genes (Basel). 2021 Feb 19;12(2):288. doi: 10.3390/genes12020288 (PMC7922028; doi:10.3390/genes12020288)
Supplement: Supplementary file 1 [file genes-12-00288-s001.zip › Supplementary Material Guo et al 2021/Supplementary Material Ghd8.pdf]

*Supplementary Materials*

# Characterization of the *Ghd8* Flowering Time Gene in a Mini-Core Collection of *Miscanthus sinensis*

**Zhihui Guo, Meilan Xu, Hironori Nagano, Lindsay V. Clark, Erik J. Sacks and Toshihiko Yamada***

***Correspondence:**

Toshihiko Yamada

yamada@fsc.hokudai.ac.jp

**SUPPLEMENTARY DATA**

Supplementary Figure S1 &S2; Supplementary Tables S1 &S2

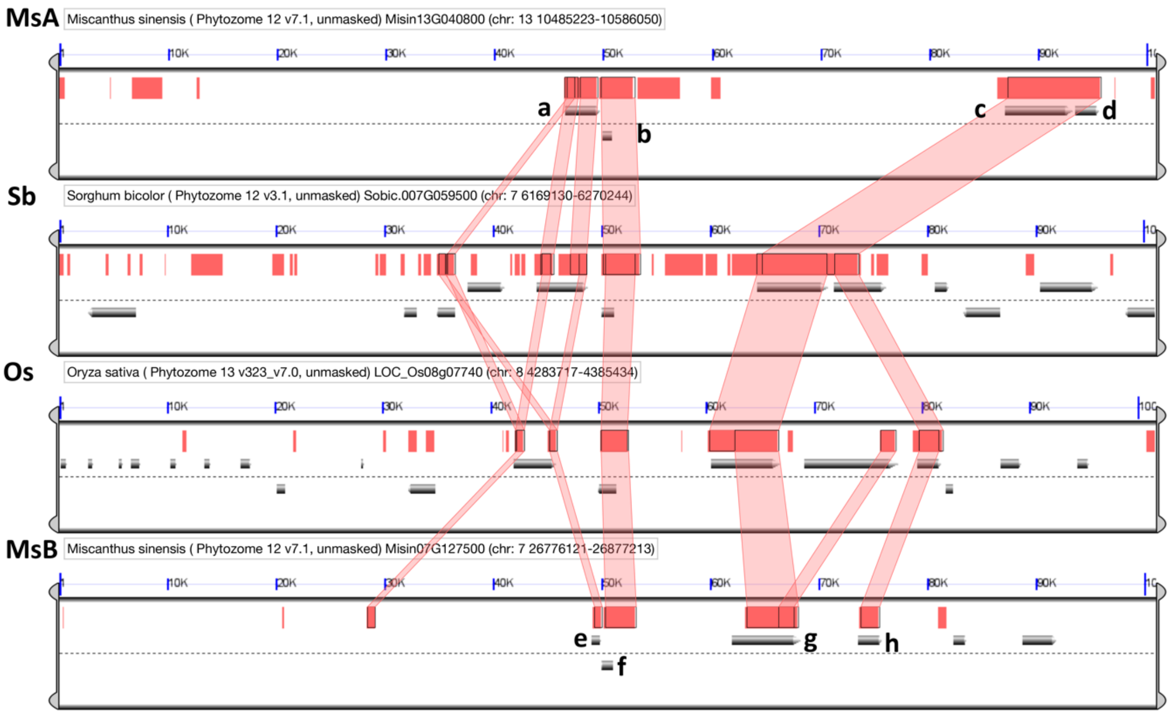


**Figure S1.** Chromosome organization of *Ghd8* gene orthologous regions (100 kbp) from *Oryza sativa*, *Sorghum bicolor*, *Miscanthus sinensis*. Only high-scoring sequence pairs (HSPs) between adjacent regions are drew in the red boxes. The dashed line in the middle of each region represents the division between the top (5′ on left) and the bottom (5′ on right) strand. The full gene models are drawn as gray arrows directly above or below this line. Colinear genes within the aligned region are connected by red lines. a-h represent colinear genes among *Oryza sativa*, *Sorghum bicolor*, *Miscanthus sinensis* (Misin13G040700- Misin13G041000, MsA; Misin07G127400-Misin07G127700, MsB) including *MsiGhd8A* (Misin13G040800, b) and *MsiGhd8B* (Misin07G127500, f), corresponding to Table S2.

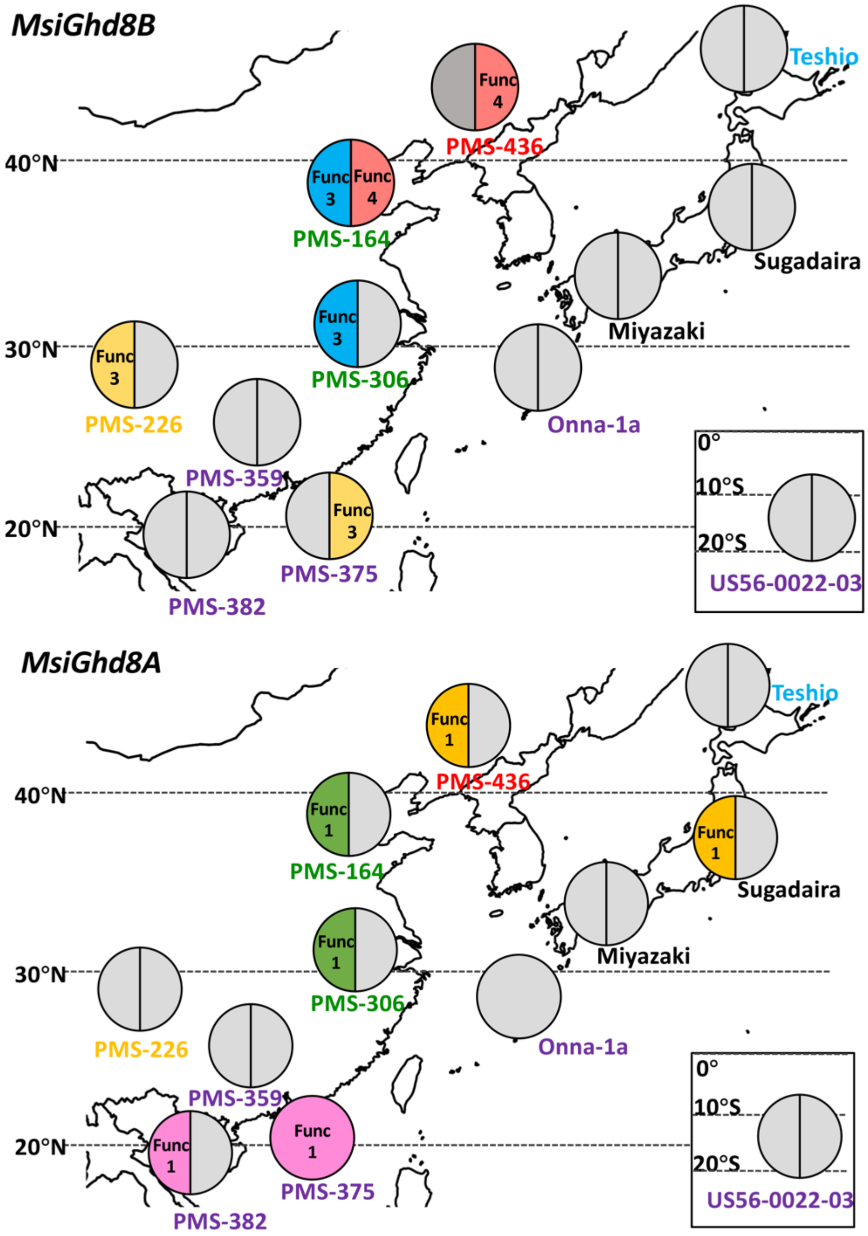


**Figure S2.** Geographical distribution of *MsiGhd8A* and *MsiGhd8B* alleles in *Miscanthus sinensis*. Pie charts with one to two sections represent the number of detected alleles. Func: putatively functional alleles, corresponding to the names in Figure 2. Pie charts with both the same color and Func number represent the same allele; alleles that were observed only once have a light gray background. Accessions' names were colored to represent *M. sinensis* genetic groups previously described by Clark et al. [49,50]; Sugadaira and Miyazaki were changed from yellow to black for making the map clear.

**Table S1 (Excel):** A summary of polymorphic sites in *MsiGhd8* open reading frames (ORFs) from 12 *Miscanthus sinensis* accessions. Cells with light blue represent single nucleotide variants (SNVs)/insertions relative to *MsiGhd8* (Misin13G040800 and Misin07G127500) in the *Miscanthus sinensis* v7.1 genome [41]. SNVs located in the domain were highlighted in the red. The A or B prefix with numbers in the right column were the protein types. Cells with different colors in the right column represent different variant types that occurred in more than one accession; variant types that were observed only once have a gray background, corresponding to Figures 2 and 3.

**Table S2**. Colinear genes near orthologs of *Ghd8* in *Miscanthus sinensis*, *Sorghum bicolor*, *Oryza sativa*

| **Colinear genes** | **Locus ID in *Miscanthus sinensis**** | **Locus ID in *Sorghum bicolor*** | **Locus ID in *Oryza sativa*** | **Putative function** |
|---|---|---|---|---|
| a, e | Misin13G040700<br>Misin07G127400 | Sobic.007G059400 | LOC_Os08g07730 | Transferase family protein |
| b, f | Misin13G040800<br>Misin07G127500 | Sobic.007G059500 | LOC_Os08g07740 | CCAAT-box-binding transcription factor |
| c, g | Misin13G040900<br>Misin07G127600 | Sobic.007G059600 | LOC_Os08g07760 | BRASSINOSTEROID INSENSITIVE 1-associated receptor kinase 1 precursor |
| d, h | Misin13G041000<br>Misin07G127700 | Sobic.007G059700 | LOC_Os08g07790 | CRS2-associated factor 2, mitochondrial precursor |

* Gene Locus IDs in *Miscanthus sinensis*, *Sorghum bicolor*, *Oryza sativa* are from Phytozome v13 (https://phytozome-next.jgi.doe.gov).
